# Cord blood porphyrin analysis in neonates at risk of inheriting protoporphyria: An observational cohort study

**DOI:** 10.1111/bjh.20252

**Published:** 2025-07-09

**Authors:** Danja Schulenburg‐Brand, Russell Peek, Lucy Bentley, Rebecca Swingler, Yana T. Pavlova, Robert S. Dawe, Victoria A. McGuire

**Affiliations:** ^1^ Cardiff Porphyria Service University Hospital of Wales Cardiff UK; ^2^ School of Medicine Cardiff University Wales UK; ^3^ Gloucestershire Hospitals NHS Foundation Trust Gloucester UK; ^4^ Three Counties Medical School University of Worcester Worcester UK; ^5^ Scottish Cutaneous Porphyria Service, Photobiology Unit University of Dundee & NHS Tayside, Ninewells Hospital & Medical School Dundee UK

**Keywords:** cord blood analysis, cord blood porphyrin, erythropoietic protoporphyria, porphyria, protoporphyria

To the Editor,

The porphyrias are a group of eight mainly inherited conditions that result from altered enzyme activity in the haem biosynthesis pathway. Erythropoietic protoporphyria (EPP) and X‐linked erythropoietic protoporphyria (XLEPP) are characterised by acute painful photosensitivity, usually within minutes of exposure to visible light. In EPP, reduced activity of erythroid ferrochelatase, which incorporates iron into protoporphyrin‐IX to form haem, causes accumulation of metal‐free protoporphyrin in the upper dermis of the skin. EPP's prevalence is estimated to be between 9 and 23 per million in Europe^1^, ^2^ and the median age of symptom onset is 1 year.^3^ In the very rare XLEPP, gain‐of‐function mutations in the *ALAS2* gene cause aminolaevulinic acid synthase 2 upregulation resulting in increased flux through the pathway. Iron incorporation into protoporphyrin‐IX becomes rate limiting, causing elevations in both metal‐free and zinc‐chelated protoporphyrin.^4^

The interaction between protoporphyrin and visible light in the dermal vasculature causes severe skin pain. Porphyrins absorb light maximally around 400–410 nm. This generates chemically unstable molecules that form reactive oxygen species, which cause cell damage.^5^

Jaundice, unrelated to protoporphyria, is a common and usually benign clinical presentation in the first days of life. However, at high concentrations, unconjugated bilirubin is neurotoxic.^6^ The American Academy of Pediatrics recommends phototherapy as first‐line treatment for significant neonatal jaundice, using light at a wavelength of around 475 nm, in the blue part of the visible spectrum^7^ where bilirubin maximally absorbs energy. Neonatal phototherapy can be delivered using a range of different light sources, including, for example, halogen bulbs, fluorescent tubes or light‐emitting diodes (LEDs). The bandwidth of emitted light varies between devices and may include the 400–410 nm bandwidth where porphyrins maximally absorb light energy.^8^

Given the potential for visible light to precipitate painful skin reactions in babies with protoporphyria,^9^ planned urgent cord blood porphyrin testing at birth of neonates at risk of inheriting protoporphyria may indicate whether special precautions need to be taken, should phototherapy be required.

There are very few reports in the literature regarding the use of either cord or peripheral venous blood porphyrin analysis at birth to identify infants who have inherited protoporphyria.^10^, ^11^ As the age of protoporphyria symptom onset is mostly from 1 year,^3^ biochemical testing is seldom undertaken in neonates and the diagnostic utility of cord blood erythrocyte porphyrin testing is unknown.

The turnaround time and blood volume required to perform plasma porphyrin, total erythrocyte porphyrin (TEP) and zinc‐chelated and metal‐free protoporphyrin analysis makes cord blood a convenient alternative to peripheral venous whole blood or genetic analysis. However, the majority of porphyria specialist laboratories are unlikely to have derived cord blood‐specific reference intervals. In addition, there is no published guidance for managing neonates with protoporphyria should jaundice requiring treatment occur.

The aim of our study was to derive a cord blood‐specific reference interval for TEP, to retrospectively review the use of cord blood porphyrin testing in two specialist porphyria laboratories in the United Kingdom and to correlate the results with clinical outcome.

From 20 samples, the full‐term newborn cord blood TEP reference interval was demonstrated to be 1.2–3.1 μmol/L of red blood cells (RBCs) which is higher than the two laboratories' adult TEP venous reference interval of <1.7 μmol/L RBC. See Data S1 for a detailed method description.

All cases of cord blood porphyrin analysis performed between 2002 and 2024 were identified, and patients followed up in the laboratories' Porphyria Centres and for whom reliable clinical information was available were included in the retrospective analysis. Outcome data included a confirmed biochemical and genetic diagnoses of EPP or XLEPP and the presence or absence of acute photosensitivity following exposure to visible light as reported during routine clinical follow‐up. All patients or parents, where clinical information was available, provided informed consent for publication.

Nine cases of cord blood TEP analysis were performed between 2002 and 2024; clinical information was available for seven patients (Table 1). Patients 1, 2, 3 and 4 had either no or very small borderline plasma porphyrin peaks and patients 2, 3 and 4 had normal TEP when judged against the cord blood‐specific reference interval. At the time of publication, none of these children, some now adults, have developed photosensitivity.

**TABLE 1:** Cord blood porphyrin results at birth.

Patient (gender)	Birth year	Cord blood plasma porphyrin		Cord blood total erythrocyte porphyrin (μmol/L RBC)^a^	Blood Zn chelated proto (%)	Indication for testing	Mutation analysis	Clinical outcome: acute photosensitivity at time of publication
1 (M)	2002	No peak		Not performed	87%	Mother EPP	Not performed	Asymptomatic
2 (M)	2003	Small borderline peak 630 nm		2.6^b^	78%	Mother EPP	Not performed	Asymptomatic
3 (F)	2019	Small borderline peak 630 nm		1.4	60%	Mother EPP	Not performed	Asymptomatic
4 (M)	2019	No peak		2.1	72%	Sibling EPP	*FECH* Heterozygous c.502C>T and c.315‐48T>C on same allele	Asymptomatic
5 (F)	2021	Peak 636 nm		21	15%	Sibling EPP	*FECH* Homozygous for c.502C>T and c.315‐48T>C	Strict light avoidance
6 (M)	2022	Peak 634 nm		39	56%	Mother XLEPP	*ALAS2* Hemizygous c.1706_1709delAGTG	Strict light avoidance
7 (M)	2023	Peak 626 nm		4.6	66%	Mother EPP	*FECH* c.315‐48T>C only	Asymptomatic

Abbreviations: EPP, erythropoietic protoporphyria; F, female; M, male; proto, protoporphyrin; RBC, red blood cells; XLEPP, X‐linked erythropoietic protoporphyria; Zn, zinc.

^a^ Adult total erythrocyte porphyrin peripheral whole blood reference interval <1.7 μmol/L RBC, cord blood reference interval 1.2–3.1 μmol/L RBC.

^b^ Maternal contamination was suspected as a cord blood reference range had not been derived at this time, repeat venous blood sample day 5 after birth showed no plasma peak and total erythrocyte porphyrin 2.1 μmol/L RBC.

Patient 5 had a large plasma porphyrin peak at 636 nm, markedly increased erythrocyte metal‐free protoporphyrin and TEP concentration of 21 μmol/L RBC in keeping with a diagnosis of EPP. Mutation analysis confirmed recessive EPP, with both a pathogenic variant c.502C>T and low expression intronic variant c.315‐48T>C on both *FECH* alleles.

Patient 6 had a large plasma peak, TEP of 39 μmol/L RBC with elevations in both erythrocyte metal‐free and zinc‐chelated protoporphyrin at birth. XLEPP was genetically confirmed weeks later. Similarly to patient 5 (Table 2), his TEP has increased over time.

**TABLE 2:** Follow up biochemical testing.

Patient	Diagnosis	Age	Total erythrocyte porphyrin (μmol/L RBC)	Mutation analysis
2	Unaffected	Birth	2.6^a^	Not performed
		Day 5	2.1	
4	EPP carrier	Birth	2.1^a^	*FECH* Heterozygous c.502C>T and c.315‐48T>C on same allele
		23 months	1.8	
5	EPP	Birth	21^a^	*FECH* Homozygous c.502C>T and c.315‐48T>C
		10 months	93	
		14 months	125	
		2 years 6 months	98	
		2 years 10 months	112	
		3 years 4 months	108	
		3 years 8 months	77	
		3 years 9 months	91	
6	XLEPP	Birth	39^a^	*ALAS2* c.1706_1709del
		9 months	77	
		11 months	95	
		16 months	104	
		23 months	100	
7	Low expression *FECH* allele carrier	Birth	4.6^a^	*FECH* c.315‐48T>C
		Day 1	5.2	
		4 months	3.1	

*Note*: Adult total erythrocyte porphyrin peripheral whole blood reference interval <1.7 μmol/L RBC, cord blood reference interval 1.2–3.1 μmol/L RBC.

Abbreviations: EPP, erythropoietic protoporphyria; RBC, red blood cells; XLEPP, X‐linked erythropoietic protoporphyria.

^a^ Cord blood, all other samples peripheral venous whole blood.

Patient 7's plasma porphyrin and TEP concentration (consisting mostly of zinc‐chelated porphyrin) were modestly raised compared to the neonatal TEP cord blood reference interval (Table 1) and confirmed on repeat peripheral venous sampling 24 h later (Table 2). The patient was not anaemic (haemoglobin 152 g/L, normal 135–195). Mutation analysis later revealed carrier status for the *FECH* low expression allele only. The modestly abnormal TEP persisted at 4 months but did not increase (Table 2). He remains asymptomatic at time of publication at age of 18 months. Cases 4, 5 and 6 are described in more detail in Data S2.

Badcock in 1996^10^ described an elevated metal‐free erythrocyte protoporphyrin 13 times and plasma total porphyrin 89 times the upper limit of a normal in an infant born to a father with EPP. No photosensitivity symptoms were reported; however, the baby was only followed up for 6 months. Hanneken et al.^11^ reported a series of 17 newborn babies with first‐degree EPP relatives. All 17 had normal cord blood TEP at birth as judged by their peripheral venous reference interval. Thirteen children were followed up for a median duration of 9.9 years, with one developing typical acute photosensitivity symptoms with a confirmed biochemical diagnosis of EPP (TEP 65 μg/dL normal <60) at age 6. Cord blood analysis therefore failed to identify this affected child at birth.

In our case series, patients 1, 2, 3 and 4 had no significant porphyrin abnormalities at birth when judged against the derived cord blood TEP reference interval, and none have developed acute photosensitivity in later life. Patients 5, 6 and 7 had abnormal cord blood plasma porphyrin and TEP at birth. Patient 5 has double homozygous EPP, patient 6 has XLEPP, but patient 7 is a carrier of the *FECH* low expression variant only. Despite the elevated TEP at birth, it consisted mostly of zinc‐chelated protoporphyrin, and so photosensitivity would not be expected.

The majority of EPP patients have a pathogenic *FECH* variant on one allele that markedly decreases ferrochelatase activity in addition to a low expression intronic variant (c.315‐48C) in *trans*. Only about 4% of EPP patients display true recessive inheritance with biallelic pathogenic *FECH* variants. In EPP families, it is more common to see affected siblings rather than parent‐to‐child transmission in contrast to XLEPP, where multigenerational disease is more common but without affected father‐to‐son transmission.^4^ Recessive and X‐linked EPP are generally characterised by higher concentrations of total erythrocyte porphyrin (TEP) and more severe skin sensitivity.

Patients 5 and 6 had grossly abnormal cord blood biochemistry but have recessive EPP and XLEPP respectively. We observed a significant increase in TEP concentrations in the early years of life in both patients. It is plausible that patients with one *FECH* pathogenic variant and the low expression variant *in trans* and with mild phenotypes may have normal or only borderline erythrocyte protoporphyrin results at birth, but with increases in protoporphyrin over time which later manifests clinically, as observed by Hanneken.^11^ This may explain the first clinical manifestations in later childhood.

Sandgren et al.^9^ recently described the case of a newborn baby who was not known to be at risk of inheriting a protoporphyria and received only 1–2 hours of phototherapy. Within hours, he developed an erythematous rash in the distribution of the phototherapy and developed acute liver failure, peripheral neuropathy and respiratory failure. He was diagnosed with XLEPP by whole exome sequencing a month later with congruent biochemistry results.

Use of phototherapy devices that emit a broad light spectrum risks infants with elevated blood protoporphyrin concentrations experiencing severe pain and distress, erythema, oedema and, if exposure is sustained, even purpura and blistering.^9^ If phototherapy is required, LED devices producing a relatively narrow bandwidth in the blue‐green part of the spectrum should be preferred. Indeed, in vitro evidence suggests that a slightly longer wavelength around 500 nm may achieve greater bilirubin degradation,^16^ although no significant difference in effectiveness was observed in vivo between phototherapy with peak emission at 459 or 497 nm.^17^

The 25 cases now published include one false‐negative case at birth as described by Hanneken^11^ and patient 7 who had elevated TEP despite being a carrier only. This demonstrates the importance of additionally analysing the metal‐free and zinc‐chelated protoporphyrin which confirmed the majority of protoporphyrin to be zinc‐chelated.

Although cord blood TEP may identify affected newborns, we do not propose newborn cord blood TEP analysis as a diagnostic test for protoporphyria at birth. Instead, we propose analysis of cord blood erythrocyte porphyrins to identify infants at risk of acute painful photosensitivity due to elevated concentrations of metal‐free protoporphyrin, should phototherapy for neonatal jaundice be required in the first few weeks of life. Infants who are at the highest risk of inheriting protoporphyria include siblings of known EPP patients, babies where both parents have EPP or one parent has EPP and the other is known to be a carrier of the low expression variant, and babies of parents with XLEPP, unless the father is affected and the fetus is known to be male. Furthermore, infants at risk of inheriting protoporphyria who have normal erythrocyte porphyrin results at birth should be reinvestigated for protoporphyria if photosensitivity manifests clinically at an older age.

These cases are rare, results are difficult to interpret and management is challenging. Where at‐risk pregnancies are identified, a multidisciplinary approach is advised, including discussion with a porphyria centre and a specialist porphyrin laboratory with expertise in analysing these samples.

## AUTHOR CONTRIBUTIONS

DS‐B and VAM conceived the idea. DS‐B, RP, RS, RSD and VAM collected data. LB and YTP performed the analysis. DS‐B wrote the paper. VAM, RP, RS, RSD, YTP and LB reviewed and made additions to the paper. DS‐B and RP responded to the reviewers. All authors approved the final version of the paper.

## CONFLICT OF INTEREST STATEMENT

The authors have no competing interests.

## Supporting information


Data S1.

Data S2.
