# Supplementary material for: Cord blood porphyrin analysis in neonates at risk of inheriting protoporphyria: An observational cohort study
Source: Br J Haematol. 2025 Jul 9;207(3):1148–51. doi: 10.1111/bjh.20252 (PMC12436226; doi:10.1111/bjh.20252)
Supplement: Supplementary file 1 — Data S1. [file BJH-207-1148-s001.docx]

**Supplement 1 – Method used to determine the total erythrocyte porphyrin (TEP) reference interval**

Twenty anonymised routinely received blood bank EDTA cord blood samples from full term newborn infants were analysed in one of the laboratories to determine a cord blood total erythrocyte porphyrin reference interval using an FL6500 fluorimeter (PerkinElmer). All the samples had a haematocrit of >0.39L/L. Non-parametric statistical analysis was performed to determine the 2.5^th^ and 97.5^th^ centiles using Microsoft Excel. The analytical coefficient of variation for the TEP assay at a mean concentration of 2.9umol/L RBC was 10.9%. The reference interval study was registered as a service improvement project with the Cardiff and Vale University Health Board (reference CDT/2023-24/02). Ethical approval was not required.

Both laboratories are accredited to ISO 15189 by the United Kingdom Accreditation Service, use the same methodology for semi-quantitative plasma porphyrin and quantitative TEP analysis [12,13] and subscribe to two international porphyrin external quality assessment schemes.

Ideally a minimum of 120 normal individuals should be selected to derive reference intervals to obtain limits with 90% certainty [14], but this is very challenging in routine service porphyria laboratories given the limited access to cord blood and the staff and time resource required to analyse TEP. The cord blood reference interval for TEP is specific to the method employed by our two laboratories [13].

A cord blood relative fluorescence unit reference interval for semiquantitative plasma porphyrin analysis was not derived as this would be fluorimeter specific due to the assay being performed without a standard. The instability of plasma porphyrin [15] makes the use of routinely collected newborn cord blood EDTA samples to derive a plasma porphyrin reference interval inappropriate.
