# Supplementary material for: Cord blood porphyrin analysis in neonates at risk of inheriting protoporphyria: An observational cohort study
Source: Br J Haematol. 2025 Jul 9;207(3):1148–51. doi: 10.1111/bjh.20252 (PMC12436226; doi:10.1111/bjh.20252)
Supplement: Supplementary file 2 — Data S2. [file BJH-207-1148-s002.docx]

**Supplement 2 -Clinical case descriptions**

Patients 4 and 5 are siblings of an index older patient who was diagnosed with EPP after presenting with acute photosensitivity at age 30 months. Patient 4 had repeat testing at 23 months of age (Table 2) which showed borderline TEP results when compared to the peripheral venous reference interval. He was found to be a carrier of both the pathogenic variant and low expression intronic variant on the same allele. He remains asymptomatic at the age of 6.

Patient 5, born prematurely at 34 weeks had cord blood results consistent with EPP. Her TEP progressively increased with age (Table 2). An early diagnosis has allowed strict photoprotective measures since birth.

Patient 6 is notable as the first published report of a pregnancy and cord blood analysis in a baby born of a mother with known XLEPP. Patient 6’s 29-year-old mother experienced photosensitivity since the age of 2 and was diagnosed biochemically and genetically confirmed to have *ALAS2* c.1706_1709delAGTG gain-of-function mutation at the age of 18. The maternal grandmother, with less severe photosensitivity, and maternal great grandfather who had been photosensitive since the age of 5, were both genetically confirmed to have XLEPP. The mother remained well with regards to bone health and liver surveillance. She developed significant idiopathic thrombocytopaenia during the pregnancy which did not respond to treatment with steroids and immunoglobulin, necessitating a general anaesthetic for planned Caesarean section.

As the condition is X-linked, offspring of an affected mother have a 50% chance of inheriting XLEPP, although penetrance in females is variable. A multi-professional team prospectively devised a postnatal management approach for the infant. Immediate, rather than delayed, umbilical cord clamping and early feed support were planned to reduce the likelihood of developing jaundice requiring treatment. Urgent TEP analysis with a turnaround time of less than 24 hours was undertaken on cord blood collected at birth. If jaundice occurred and required treatment, a light emitting diode (LED) phototherapy device with narrow spectrum emission between 450 and 475nm and minimal emission at 400nm would be used, with close monitoring for signs of distress. An experienced neonatologist would consider adjusting phototherapy thresholds or using alternative treatments for jaundice should signs of distress occur.

Patient 6 was born at term by planned caesarean section. He weighed 3.87kg and had a normal newborn physical examination. Cord blood TEP concentration available within 24hrs of birth was 39µmol/L RBC suggesting he had inherited XLEPP (later confirmed genetically) and would be photosensitive. Strict photoprotection was implemented from birth. Effective breastfeeding was established before discharge. The baby remained well in the post-natal period and did not require treatment for jaundice. Similarly to patient 5, he has demonstrated a significant increase in TEP in the first few years of life. At 4 years of age, the child remains well.
